# Experiences with simultaneous use of contraception and the vaginal ring for HIV prevention in sub-Saharan Africa

**DOI:** 10.1186/s12905-021-01321-5

**Published:** 2021-04-23

**Authors:** Jonah Leslie, Flavia Kiweewa, Thesla Palanee-Phillips, Katherine Bunge, Felix Mhlanga, Betty Kamira, Jared Baeten, Ariana Katz, Sharon Hillier, Elizabeth Montgomery, for the MTN-020/ASPIRE Study Team, Jared Baeten, Thesla Palanee-Phillips, Elizabeth Brown, Lydia Soto-Torres, Katie Schwartz, Bonus Makanani, Francis Martinson, Linda-Gail Bekker, Vaneshree Govender, Samantha Siva, Zakir Gaffoor, Logashvari Naidoo, Arendevi Pather, Nitesha Jeenarain, Gonasagrie Nair, Thesla Palanee-Phillips, Flavia Matovu, Nyaradzo Mgodi, Felix Mhlanga

**Affiliations:** 1 Women’s Global Health Imperative, RTI International, San Francisco, CA USA; 2 Makerere University-Johns Hopkins University Research Unit, Kampala, Uganda; 3 Wits Reproductive Health and HIV Institute (Wits RHI), Hillbrow, South Africa; 4 Department of Obstetrics, Gynecology and Reproductive Sciences, University of Pittsburgh, Pittsburgh, PA USA; 5 UZ-UCSF Collaborative Research Programme, Harare, Zimbabwe; 6 Departments of Global Health, Medicine, and Epidemiology, University of Washington, Seattle, WA USA; 7 Magee-Womens Research Institute, Pittsburgh, PA USA

**Keywords:** HIV, Contraception, Qualitative research, Vaginal ring, Africa South of the Sahara, Clinical trial

## Abstract

**Background:**

Clinical trials have found that a monthly dapivirine vaginal ring was well-tolerated and reduced HIV-1 risk among women in sub-Saharan Africa. However, in order for the ring or other novel prevention methods to have optimal impact, it is necessary to understand and address women’s challenges to uptake and adherence. This paper provides insight into a few key challenges noted by women using the ring and contraceptives simultaneously.

**Methods:**

The qualitative portion of the MTN-020/ASPIRE study consisted of data collection using single in-depth interviews, serial in-depth interviews, and focus group discussions, conducted with 214 participants across 15 sites in Malawi, South Africa, Uganda and Zimbabwe. A coding team used qualitative analysis software to identify themes within the interviews.

**Results:**

The primary qualitative themes among participant data pertained to side effects. Participants reported negative side effects related to menses, in some cases attributing these effects to their contraceptives and in others to the vaginal ring. Participants also expressed concern over the long-term impact of contraception and ring use on fertility, including the reversibility of the contraceptive, especially among nulliparous women.

**Conclusions:**

Women’s attitudes toward contraceptives can impact their willingness to concurrently use and adhere to a novel HIV prevention product. To optimize the potential of both prevention products, researchers should pre-emptively address concerns about contraceptive impact on fertility and counsel women about the expected side effects of contraceptives versus the ring.

*Clinical trials identifier* NCT01617096. Registered on 6-12-2012 at clinicaltrials.gov https://clinicaltrials.gov/ct2/show/NCT01617096

**Supplementary Information:**

The online version contains supplementary material available at 10.1186/s12905-021-01321-5.

## Background

Women of childbearing age in sub-Saharan Africa continue to face disproportionate risk of HIV-1 acquisition compared to men and their global counterparts [1]. Two clinical trials (MTN-020/ASPIRE and IPM 027/The Ring Study) reported results showing that a monthly dapivirine vaginal ring was well-tolerated and reduced HIV-1 risk among women in sub-Saharan Africa [2, 3]. Open label extension studies of the vaginal ring have recently completed, confirming the safety profile of the ring as well as demonstrating a reduction in expected HIV-1 incidence [4].

As was demonstrated in the Phase III and open label extension studies, the efficacy of the ring relies on its consistent use. In the MTN 020/ASPIRE trial, women were required to use a highly effective method of contraception throughout their participation, as the effect of the drug and ring on the fetus or pregnancy was unestablished [2]. In order for the ring or other novel prevention methods to have optimal impact on reducing risk of HIV infection, it is necessary to address factors that influence women’s fears and concerns [5], as well as other challenges to uptake and adherence. Concurrent use of an HIV prevention product and a contraceptive is one such challenge. Widespread adoption of the dapivirine ring requires that issues around users’ fear of contraceptive side effects be recognized in the context of adherence to the ring.

MTN020/ASPIRE participants were provided with a choice of contraceptive pills, injections (DMPA, Net-En), implants (Jadelle, Nexplanon), or the copper IUD, either directly at the trial sites or through referrals within the public sector. They were also offered contraceptive counseling. This qualitative methods paper provides insight into some of the key challenges noted by users of the ring and contraceptives, and addresses implications of these challenges for the future success of the dapivirine ring.

## Methods

### Population and study design

In the full ASPIRE trial, a total of 2629 women were enrolled across 15 sites in Malawi, South Africa, Uganda, and Zimbabwe [2]. The ASPIRE study design, population and procedures are published elsewhere, but briefly, women were aged 18–45 and were HIV-negative, nonpregnant or breastfeeding and sexually-active at enrollment [2]. They were followed for a minimum of 12 months from 2012 to 2015, with a qualitative component conducted at six sites spanning from 2013 to 2015 [6]. The qualitative portion consisted of single in-depth interviews (IDI), serial IDIs (SIDI), and focus group discussions (FGD), conducted with a total of 214 participants composed of those who were randomly preselected from ASPIRE participants, those who were purposively selected as “special cases” based on their unique adherence experiences, and those who discontinued early because of HIV seroconversion [6]. SIDI allowed for the study of longitudinal themes and increased rapport between interviewers and respondents. IDI targeted trial participants who had discontinued ring use, typically due to seroconversion, pregnancy, or grade 3 or 4 adverse events. FGD were conducted to examine group perceptions of the ring. The SIDI and IDI interview guide is provided as Additional File 1. The FGD interview guide is provided as Additional File 2. SIDI were conducted near the conclusion of participants’ third and twelfth months in the study, and around the time of their Product Use End Visit (PUEV). FGD were conducted at or around participants’ PUEVs. The timing of single IDI varied.

Modern contraception was a requirement for trial eligibility. The study included interviews with participants who initiated or changed contraception methods prior to enrollment, as well as those already using effective methods. Contraceptive counseling, covering the expected efficacy, use, and mechanism of each method, was offered at screening and enrollment visits, as well as all follow-up visits. Participants were also counselled on potential side effects and ways that these side effects could be clinically managed. Counselling was typically provided by nurses, with support by physicians and pharmacists. When experiencing side effects, participants were provided with medication or guidance on changing methods, which were provided at no cost.

### Data collection and analysis

Sixteen Institutional or Ethics Review Boards, representing each qualitative data collection site approved the study. All participants provided written informed consent. Interviews were conducted in local languages by trained social scientists using semi-structured guides covering participants’ motivation to join the trial, perceptions of HIV risk, trial experiences, male partner perceptions, and the ring’s effect on sex. Participants were further queried about how their participation in ASPIRE influenced their contraceptive use and their experiences using the ring during menses, with probes to explore their experience of side effects. Interviews were audio-recorded, transcribed verbatim, and translated into English before being uploaded into NVivo 11 qualitative analysis software for coding. Both deductive and inductive coding was employed, with checks to maintain 90% intercoder reliability on 10% of interviews. The coding team discussed themes over a 14 month period. Themes surrounding contraception concerns were examined by a subset of analysts and coauthors. Specifically, the following codes were analyzed for this paper: Contraception, Discontinuation, Removal, Side Effects, Staff, Negative Influence, and Positive Influence. Because the area of contraceptive challenges was not a key objective of the ASPIRE study, the topics addressed in this paper arose unevenly across participant interviews. Therefore, descriptors of specific quantitative prevalence have been avoided in favor of focusing on the salience of described themes, with an emphasis on exemplary quotes as preferred for qualitative reporting of this nature [7].

## Results

Table 1 displays participant characteristics of the qualitative subset of the study population. Participants’ ages ranged from 18 to 24, with a median age of 26 years. 73% had completed at least some secondary school. 55% were not married.

**Table 1:** Participant characteristics

	QUAL sample
Participants enrolled, N	214
Currently married	
Yes	96 (45%)
No	118 (55%)
Highest level of education	
No schooling	5 (2%)
Primary school (partial and complete)	45 (21%)
Secondary school (partial and complete)	156 (73%)
Attended college or university	8 (4%)
Primary partner knows of participation in the trial	
Yes	153 (72%)
No	60 (28%)
Not sure	–
Primary partner knows has been asked to use ring	
Yes	125 (59%)
No	88 (41%)
Not sure	–
Primary partner HIV status	
HIV positive	4 (2%)
HIV negative	125 (59%)
Participant does not know	84 (39%)
Had same primary partner for last 3 months	
Yes	206 (97%)
No	7 (3%)
Current method of contraception	
Intrauterine device	25 (12%)
Oral contraceptives	14 (7%)
Injectable contraceptives	121 (57%)
Depot medroxyprogesterone acetate (DMPA)	94 (78%)
Norethisterone enanthate (NET-EN)	27 (22%)
Hormonal implant	50 (23%)
Sterilization	5 (2%)
Age, years	
Median (range)	26 (18–42) [213]

The primary qualitative themes about contraceptive use pertained to side effects, both immediate effects on health and comfort, and fears of long-term effects on fertility.

Participants across multiple sites reported experiencing side effects related to menses, including spotting or heavy bleeding, prolonged menses, irregular menstruation, and other menses-related issues, such as weight gain. Some participants attributed these side effects to their contraceptives, and they reported their issues to staff members or requested to change their method. Despite high retention in the trial, the side effects that participants experienced led them to consider discontinuation from ASPIRE.

I had a problem with the prevention. I was a person who did not like to use prevention. Now that I was in the study and using the ring, it was a requirement to use prevention. I ended up using it because it was a requirement that while I’m here I have to use it. When I started using it, it did not treat me well. I did not know whether I should quit the study or what because I menstruated for a long time. [Johannesburg, South Africa]

Furthermore, participants experienced side effects that may have been caused by contraceptives but were attributed to the study ring. Several women reported removing their rings after experiencing such side effects and choosing not to reinsert before their next study visit. When participants brought these issues to the attention of staff during clinic visits, staff often explained the side effects were caused by contraceptives, but some women remained skeptical.

For me when we had just started using the ring, I started using the IUCD as well. I bled so much because I spent three months bleeding. And whenever I would lift anything heavy, I would feel pain in the lower abdomen. I got scared and thought that maybe it is the ring that had caused that bleeding. I wondered what I could do but I never removed the ring. But almost every week I would come to the study clinic and tell the health workers that I was feeling a lot of pain and was bleeding. I asked them if it was the ring causing that, but they would tell me it is not the ring but the IUCD for family planning… [Kampala, Uganda]

Even when side effects were not experienced directly by participants, possible side effects from contraceptives were still a concern. Contraceptives’ impact on fertility was a particular concern. Participants expressed fears that the contraceptive they used during the study would make them infertile or make conception difficult later on, especially if the woman had not yet had children.

People tell me if you are on contraception and you’ve never had a baby before it becomes complicated to have a baby in the future. Even elders say if you are [on] contraception without having borne a child then you won’t have babies at all” [Durban, South Africa]

In some cases, fears about contraception and fertility were based in misunderstanding of the reversibility of contraceptive methods.

It troubled me because… some said Depo is a problem in that when you want to get pregnant it will be a challenge and you can spend even 4 or 5 years without getting pregnant while trying to get pregnant. [Harare, Zimbabwe]

Participants further indicated that these fears could be exacerbated by the knowledge that two distinct products were in use simultaneously.

You might always be thinking about having two things inside your body; it makes you be worried all the time…. You might be worried that having those two things might cause you some problems. [Kampala, Uganda]

## Discussion

Participant narratives revealed that negative side effects caused by or perceived to be caused by contraceptives could be a barrier to ring adherence. Changes in bleeding patterns, weight gain, and other effects led them to consider discontinuing study participation. Even when such effects were not experienced personally, fear of side effects generated by listening to other participants sometimes led women to question their own continued use of HIV preventative products [5, 8]. Future participants in clinical trials or users of new HIV prevention methods outside the study environment could be prone to discontinuing HIV prevention measures if they associate known contraceptive side effects with the prevention measure. This may be of particular importance if they start using the two products simultaneously.

Actual and perceived contraceptive side effects influencing acceptance could also pose a barrier to consistent use of multipurpose technologies (MPTs) that are currently under investigation for pregnancy and HIV prevention. In ASPIRE, women cited irregular menses, abdominal pain, and other side effects as a barrier to their continued ring use, in a context where the flexibility of switching contraceptive methods was readily available. In the full study, 34% of participants had switched contraceptive methods at 12 months [5]. Because MPTs inherently link pregnancy and HIV prevention into a single product, nonadherence due to side effects for one indication would directly impact adherence to the other. Detailed counselling for these side effects could reduce discontinuation of MPT use.

Women indicated a concern that the ring or contraceptive might continue to act years after discontinuation or cause permanent damage to their fertility. This sentiment echoes results from other studies [9, 10]. Sub-Saharan Africa is a setting in which infertility can have far-reaching effects on women’s social, economic, and physical health [11]. Respectively, testing and roll-out of HIV prevention methods like the dapivirine ring or future MPT should pre-emptively address the importance of fertility to users and the community.

Use of a reliable form of contraception among participants in this trial was a requirement because of the investigational nature of dapivirine, and unknown effect of the ring during pregnancy. The Microbicide Trials Network has two trials in development to evaluate the safety of the dapivirine ring among pregnant (MTN-042) and breastfeeding (MTN-043) women [12]. Oral PrEP use in pregnancy has been supported in some HIV endemic settings and is believed to be safe [13]. Preliminary analysis of pregnancy and infant outcomes in the Phase III dapivirine ring trials indicates that the ring is not associated with adverse effects in early pregnancy [14]. However, until more definitive research results are released, the challenges that contraceptives pose when evaluating HIV prevention tools will need to be addressed for concurrent use of HIV PrEP technologies and contraceptives.

## Limitations

The topic of contraception challenges was not a key objective of the ASPIRE study and was not systematically queried. However, the interview guides and codes did allow for a targeted secondary analysis of themes surrounding contraception, reproductive health, and associated barriers to ring use. The sample size of interview passages also limits the conclusiveness of thematic differences seen between sites, so site comparisons have not been explored.

Furthermore, many women in the study started or switched contraceptive methods upon joining the study [15]. In a typical environment, women considering using the ring may have an established contraceptive routine already. As a result, they could be less likely to attribute familiar contraceptive side effects to the ring.

## Conclusion

Women’s attitudes toward and experience with contraceptives can impact their willingness to concurrently adhere to a novel HIV prevention product like the dapivirine ring. In order to optimize the potential of both prevention products, researchers should pre-emptively address concerns about contraceptive impact on fertility, provide reassurance that contraceptives will be reliably reversible, and counsel women about expected side effects of contraceptives versus the ring. The real-world success of the dapivirine ring could be significantly impacted by how concerns about concurrent contraceptive products are addressed.

## Supplementary Information

**Additional file 1.** In-depth interview topic guide.

**Additional file 2.** Focus group discussion topic guide.

## Data Availability

The datasets generated and/or analyzed during are not publicly available due to the need to maintain participant confidentiality.

## Abbreviations

FGD: Focus group discussions
MPTs: Multipurpose technologies
PUEV: Product use end visit
IDI: Single in-depth interviews
SIDI: Serial in-depth interviews
