# Supplementary material for: Experiences with simultaneous use of contraception and the vaginal ring for HIV prevention in sub-Saharan Africa
Source: BMC Womens Health. 2021 Apr 23;21:175. doi: 10.1186/s12905-021-01321-5 (PMC8067655; doi:10.1186/s12905-021-01321-5)
Supplement: Supplementary file 1 — Additional file 1. In-depth interview topic guide. [file 12905_2021_1321_MOESM1_ESM.pdf]

MTN-020

In-depth Interview (IDI) Topic Guide

INSTRUCTIONS for the Interviewer: How to use the IDI Guide

1. There are two levels of questions:

   - Primary interview questions: appear in **bold** text. They address the topics that you as the interviewer must ask and discuss with participants. The questions are suggestions for getting the discussion going. You are not required to read them verbatim, but they are written to ensure some consistency across IDIs. You may adapt the questions and/or ask them in a different order, depending on how the interview develops. However you will have to ensure that by the end of the interview, all the topics and key themes have been covered.

   - Probing topics: are indicated with a bullet. If you find that the participant provides little information in response to the primary question, these probing topics may be used to encourage further discussion. You are not required to cover every topic listed. So, depending on what has already been discussed, and the IDI context, you may ask these probes or not.

2. *Instructions/suggestions to interviewer are in italics and [brackets].*

3. Words found in (parentheses) are meant to provide wording options to interviewers to fit various situations. For example, they often provide a present or past tense verb. In these instances, the present tense verb should be used when the participant being interviewed is continuing with the study, whereas the past tense verb should be used with those participants who are exiting or have finished participating in the study.

4. The IDI guide is divided into two columns.

   - **The left-hand column** contains the research questions and probes to be used during the first IDI with serial IDI participants or during an IDI with participants who are discontinuing the product early.

   - **The right-hand column** contains the research questions and probes to be used during the repeated IDIs with serial IDI participants. Prior to conducting these IDIs, the interviewer should review notes, the debriefing report, and/or interview transcript from the participant's previous interview(s) in order to help form appropriate probes. The focus of these questions should be on changes or new issues since the previous interview.

5. The IDI guide is not meant to be used to take notes. Rather, you should use the separate notes form, where you will also insert your initials, the participant's PTID, as well as the date, start and end time of the interview.

Before starting the IDI, ensure the participant has provided written informed consent.

[***Start Recorder and Read Introduction***]: My name is _________________. Thank you again for your willingness to be a part of this discussion. I am looking forward to hearing your thoughts on the questions I will ask you. Please know there are no right or wrong answers and we welcome every opinion about the topics we will discuss, so feel free to share your thoughts, opinions, and views openly. If during our discussion, there are issues or concerns that you would like to talk about, feel free to bring them up, even if I didn't ask about them. If you have specific questions during the interview, I will take note of them and answer them directly after the interview. If I cannot answer them, I can refer you to someone who may be able to help. Before we start, can you confirm for the recorder that you have already provided written informed consent to take part in this discussion? [*Wait for oral confirmation to begin*].

As I mentioned before, the main goal of this discussion is to better understand your experience participating in ASPIRE. I want to remind you that what we discuss here will be kept confidential, and that we will not share your personal information or responses with anyone outside of the study.

Serial IDI Visit 1/Early Product Discontinuers	Repeat Serial IDI Visit(s)
**Motivations to Join/Stay in Study and HIV Risk Perception**	
**First we are going to talk about joining the study in general and your feelings about HIV…**	
**1. Why did you join the study?**<br>*Possible probing topics:*<br>• Services or benefits received while in the trial<br>• Aspects of her personal life<br>• Aspects of the community/the place where she lives	**1. Why have you continued to participate in the study?**<br>*Possible probing topics:*<br>• Services or benefits received for participating in trial<br>• Aspects of participant's personal life<br>• Aspects of the community/the place where the participant lives
**2. Tell me about your experience being part of ASPIRE.**<br>*Possible probing topics:*<br>• Likes and dislikes and reasons<br>• Experiences during study visits or at the clinic, including site specific participant engagement activities<br>• Effects of study on daily life for participant, partner(s), and others<br>• Changes in relationship with male partner(s), including decision-making ability and communication around HIV prevention	**2. Tell me about your experience being part of ASPIRE.**<br>*Possible probing topics:*<br>• Experiences during study visits or at the clinic, including site specific participant engagement activities<br>• Effects of continued study participation on daily life for participant, partner(s) and others<br>• Changes in relationship with male partner(s), including decision-making ability and communication around HIV prevention
**3. Before you joined ASPIRE, how worried were you about getting HIV?**<br>*Possible probing topics:*<br>• Reasons for level of worry<br>• How HIV worries influences decision to join ASPIRE<br>• How HIV worries influences uptake of the ring	
**4. After you joined ASPIRE, how did your worries about getting HIV change?**<br>*Possible probing topics:*<br>• Influence of behavior changes (if any) , including changes in sexual partners/practices, alcohol/substance use, or other risk behaviors<br>• Influence of study procedures (e.g. HIV testing, counseling)<br>• Influence of wearing the ring, including perceptions about protection and belief of whether its active or placebo	**3. Since joining ASPIRE, how have your worries about getting HIV changed?**<br>*Possible probing topics:*<br>• Influence of behavior changes, including changes in sexual partners/practices, alcohol/substance use, or other risk behaviors<br>• Influence of study procedures (e.g. HIV testing, counseling)<br>• Influence of wearing the ring, including perceptions about protection and belief of whether its active or placebo
**Use Experiences: Acceptability & Adherence**	
**Now we are going to talk more about your experiences using the ring during this study…**	
**[*Provide body map materials/drawing and explain activity. Use the visual and questions to discuss the questions below.*]**<br>**5. Using this drawing, describe how you think the ring works?**<br>*Possible probing topics:*<br>• Ring location while inside the body<br>• How ring protects against HIV<br>• Concept of placebo ring	

**6. Please describe how the ring feels inside your body?**<br>*Possible probing topics:*<br>• Feeling upon initial insertion and once inside<br>• Situations when she is more or less aware of the ring<br>• Other effects of the ring in her body	**4. Please describe how the ring feels inside your body?**<br>*Possible probing topics:*<br>• Feeling upon most recent insertion and once inside<br>• Situations when she is more or less aware of the ring<br>• Other effects of the ring in her body
**7. What is your opinion of the ring?**<br>*Possible probing topics:*<br>• Likes and dislikes about physical attributes, such as color, texture, and size<br>• Likes and dislikes about how it is used	**5. What is your opinion of the ring?**<br>*Possible probing topics:*<br>• Opinions about physical attributes, such as color, texture, and size<br>• Any other likes and dislikes about physical attributes<br>• Likes and dislikes about how it is used
**8. In your opinion, how well (are/were) you doing at using the ring the way you (are/were) supposed to?**<br>*Possible probing topics:*<br>• How she perceives she is supposed to use the ring<br>• Ease/difficulty wearing the ring continuously between visits<br>• How and with whom did she make decisions around ring use (e.g. male partner, family members, other participants)<br>• Influence of feedback from staff regarding how her ring looks, site level adherence performance, or other support offered on ring use	**6. How well do you think (are/were) you doing at using the ring the way you (are/were) supposed to?**<br>*Possible probing topics:*<br>• Perception of how she is supposed to be using the ring<br>• Ease/difficulty wearing the ring continuously between visits<br>• How and with whom did she make decisions around use (e.g. male partner, family members, other participants)<br>• Influence of feedback from staff regarding how her ring looked, site level adherence performance, or other support offered on ring use
**9. Tell me about any times when you took the ring out or it came out on its own, either partially or fully.**<br>*Possible probing topics:*<br>• Timing and circumstances when ring came out or was removed<br>• Instances of partner removing ring<br>• Position of her body when ring came out<br>• What did she do about it<br>• [If re-inserted outside of the clinic]was ring cleaned and how<br>• Removal reported to the clinic, why or why not.	**7. Tell me about any times when you took the ring out or it came out on its own, either partially or fully.**<br>*Possible probing topics:*<br>• Timing and circumstances when ring came out or was removed<br>• Instances of partner removing the ring<br>• Position of her body when ring came out<br>• What did she do about it<br>• [If re-inserted outside of the clinic] was ring cleaned and how<br>• Removal reported to the clinic, why or why not
**10. Tell me about a specific time when you had a challenge with the ring.**<br>*Possible probing topics:*<br>• Physical, personal (e.g. with your partner), or emotional challenge<br>• Any challenges related to alcohol/other substance use<br>• Timing and circumstances of challenge<br>• If and how challenge was resolved	**8. Tell me about a specific time when you had a challenge with the ring.**<br>*Possible probing topics:*<br>• Physical, personal (e.g. with your partner), or emotional challenge<br>• Any challenges related to alcohol/other substance use<br>• Timing and circumstances of challenge<br>• If and how challenge was resolved
**11. How (does/did) the ring affect your sexual life?**<br>*Possible probing topics:*<br>• Ring removals before or during sex<br>• Awareness of the ring during sex for you and partner<br>• Positive or negative physical or emotional changes with sex<br>• Changes in sexual practices, sexual partners, frequency, or reasons for having sex, etc.	**9. How (does/did) the ring affect your sexual life?**<br>*Possible probing topics:*<br>• Ring removals before or during sex<br>• Awareness of the ring during sex for you and partner<br>• Positive or negative physical or emotional changes with sex<br>• Changes in sexual practices, sexual partners, frequency, or reasons for having sex, etc.

____
Staff Initials / Date

**12. How (does/did) your participation in ASPIRE influence(d) your contraceptive use?**<br>*Possible probing topics:*<br>• Changes in contraceptive methods used<br>• Reasons for changes, if any<br>• Perceived influence of contraceptives on menses and fertility	**10. How (does/did) your participation in ASPIRE influence(d) your contraceptive use?**<br>*Possible probing topics:*<br>• Reasons for changes, if any<br>• Perceived influence of contraceptives on menses and fertility
**13. How (does/did) your participation in ASPIRE influence(d) your condom use?**<br>*Possible probing topics:*<br>• Changes in patterns of condom use, including ability to negotiate their use with partners<br>• Reasons for changes, if any	**11. How (does/did) your participation in ASPIRE influence(d) your condom use?**<br>*Possible probing topics:*<br>• Changes in patterns of condom use, including ability to negotiate their use with partners<br>• Reasons for changes, if any<br>• Feelings about using the ring with condoms (currently and in the future, if effective)
**14. What has been your experience with using the ring during menses?**<br>*Possible probing topics:*<br>• Perceived effect of ring on menses and/or fertility<br>• Changes in menstrual practices, reasons for the changes<br>• Changes in menstrual symptoms or cycle, feelings about these changes<br>• Ring removal or expulsion during menses<br>• Preference for use/non-use of ring during menses	**12. What has been your experience with using the ring during menses?**<br>*Possible probing topics:*<br>• Perceived effect of ring on menses and/or fertility<br>• Impacts of the ring on menstrual practices and feelings about the changes in practices, if any<br>• Changes in menstrual symptoms or cycle, feelings about these changes.<br>• Ring removal or expulsion during menses<br>• Preference for use/non-use of ring during menses
**15. Can you describe the kinds of practices you did to your vagina prior to joining ASPIRE?**<br>*Possible probing topics:*<br>• Types of practices: cleansing , application or insertion of products/substances<br>• Timing of practices: frequency (i.e. daily, weekly, etc.), before or after sex, before or after menstruation<br>• Reasons for practices<br>• Changes in practices due to study/ring use or other reasons	**13. Can you describe the current practices you do to your vagina?**<br>*Possible probing topics:*<br>• Reasons for changes or lack of changes in vaginal practices – changes due to study/ring use or other reasons
**16. [*Only ask of participants discontinuing product early*] How does the ring compare with other products that are currently used to prevent HIV or may be used in the future?**<br>*[Refer to "Product formulation for HIV prevention" visual discussion tool.]*	**14. [Only a*sk at IDI conducted upon study exit visit]* How does the ring compare with other products that are currently used to prevent HIV or may be used in the future?**<br>*[Refer to "Product formulation for HIV prevention" visual discussion tool.]*

____
Staff Initials / Date

<table>
<tr><th colspan="2">Disclosure and Partners</th></tr>
<tr><td colspan="2">Now I want to talk more about your sexual partner(s) and other people who are close to you….</td></tr>
<tr><td>17. Who have you told about your use of the ring?
Possible probing topics:
• Discussions with other study participants in the clinic, outside the clinic, and at ASPIRE events about ring adherence
• Discussions with other people about ring use: primary sex partner, other partners, family members, friends, anyone else
• Reasons for disclosure and nondisclosure
• How people found out in cases of nondisclosure, what was said, reactions and attitudes to study and ring, and the participant's feelings about the disclosure
• Influence of discussions on ring use or study participation</td><td>15. Who have you told about your use of the ring?
Possible probing topics:
• Discussions with other study participants in the clinic, outside the clinic, and at ASPIRE events about ring adherence
• Discussions with other people about ring use
• Who was told, what was discussed, reactions and attitudes to study and ring, and participant's feelings about disclosure
• Reasons for disclosure and nondisclosure
• Influence of discussions on ring use or study participation</td></tr>
<tr><td>18. What (does/did) your sexual partner(s) think about the ASPIRE study in general?
Possible probing topics:
• Partner's level of interest and involvement in the study including his level of participation in any ASPIRE events or visits to the clinic for any referrals or treatment
• Reaction of different partners, if multiple partners
• Concerns about contraceptive use requirement, if known</td><td>16. What (does/did) your sexual partner(s) think about the ASPIRE study in general?
Possible probing topics:
• Confirm if partner(s) is/are same or different from last discussion
• Change since initial disclosure/reaction
• Partner's level of interest and involvement in the study, including his level of participation in any ASPIRE events or visits to the clinic for any referrals or treatment
• Reaction of different partners, if multiple partners
• Concerns about contraceptive use requirement, if known</td></tr>
<tr><td>19. What (is/was) your sex partner's opinion of the ring?
Possible probing topics:
• Partner's likes, dislikes, concerns/worries for himself, for the participant, or for the relationship
• Role of "female-initiated" nature of the ring in his opinions
• Role of ring in introducing/aggravating any challenges in the relationship
• Partner's level of involvement in decision to use the ring
• His role in supporting or discouraging use
• Conflicts and resolution around ring use
• Impact on his sexual experience/the sexual relationship
• If multiple partners, opinion of other partners</td><td>17. What (is/was) your sex partner's opinion of the ring?
Possible probing topics:
• Partner's likes, dislikes, concerns/worries for himself, for the participant, or for the relationship
• Role of "female-initiated" nature of the ring in his opinions
• Role of ring in introducing/aggravating any challenges in the relationship
• Partner's level of involvement in decision to use the ring
• Partner's role in supporting or discouraging use
• Impact on his sexual experience/the sexual relationship
• If multiple partners, opinion of other partners</td></tr>
</table>

Staff Initials / Date

**20. What is your opinion of using the ring without telling your sex partner?**<br>*<u>Possible probing topics:</u>*<br>• How important is it to involve male partners in decisions to use the ring<br>• What has been her experience?<br>• Reasons for telling or not telling a partner, including role of fear around negative reactions/violence<br>• Partner's reactions to ring use without telling him<br>• Suggestions for engaging men.	**18. What has been your experience using the ring without telling a sexual partner?**<br>*<u>Possible probing topics:</u>*<br>• New experiences since last IDI<br>• Reasons for not telling partner, including role of fear around negative reactions/violence<br>• Partner's reactions, if any<br>• Changes in opinion of how important is it to involve male partners in decisions to use the ring<br>• Suggestions for engaging men
**Recommendations and Feasibility of Future Use**	
**We are coming towards the end of our discussion. Before we finish, I would appreciate your recommendations and thoughts to help us with future work….**	
**21. What could we (do/have done) to improve your experience in the study?**<br>*<u>Possible probing topics:</u>*<br>• Participant and partner engagement activities<br>• Study visits, study procedures, clinic<br>• Interaction with staff, counseling sessions<br>• Reducing interference with daily life (e.g. due to clinic visits or other requirements)<br>• Ideas to avoid fatigue with study participation, in general/keep participation fresh	**19. What could we (do/have done) to improve your experience in the study?**<br>*<u>Possible probing topics:</u>*<br>• Participant and partner engagement activities<br>• Study visits, study procedures, clinic<br>• Interaction with staff, counseling sessions<br>• Reducing interference with daily life (e.g. due to clinic visits or other requirements)<br>• Ideas to avoid fatigue with study participation, in general/keep participation fresh
**22. [*Only ask of participants discontinuing product early*] What could we have done to improve your experience using the ring?**<br>*<u>Possible probing topics:</u>*<br>• Couple counselling to aid with ring use disclosure<br>• Changes in design of ring: physical characteristics<br>• Insertion, removal method, frequency of ring replacement<br>• Feelings about single- or multi- purpose rings (i.e. HIV prevention only or HIV prevention and contraception), including related concerns about future fertility<br>• Instructional materials, or how the instructions were provided	**20. [Only a*sk at IDI conducted upon study exit visit]*What could we have done to improve your experience using the ring?**<br>*<u>Possible probing topics:</u>*<br>• Couple counselling to aid with ring use disclosure<br>• Changes in design of ring: physical characteristics<br>• Insertion, removal method, frequency of ring replacement<br>• Feelings about single- or multi-purpose rings (i.e. HIV prevention only or HIV prevention and contraception), including related concerns about future fertility<br>• Instructional materials, or how the instructions were provided
**23. [*Only ask of participants discontinuing product early for reasons other than seroconversion*] If the ring is found to protect from HIV, do you think that you will use it for HIV prevention?**<br>*<u>Possible probing topics:</u>*<br>• Interest in participating in a follow-up study where everybody is given an active ring (containing dapivirine)<br>• Ability to use and interest in using the ring<br>• Issues around access, storage, disposal, discreetness concerns about short term vs. long term use on fertility/health or hygiene<br>• Comfort using the ring while taking alcohol or drugs<br>• Impact of community, partners or others individuals' feelings about the ring	**21. [Only a*sk at IDI conducted upon study exit visit]* If the ring is found to protect from HIV, do you think that you will use it for HIV prevention?**<br>*<u>Possible probing topics:</u>*<br>• Interest in participating in a follow-up study where everybody is given an active ring (containing dapivirine)<br>• Ability to use and interest in using the ring<br>• Issues around access, storage, disposal, discreetness, concerns or issues with short term vs. long term use on fertility/health or hygiene<br>• Comfort with using ring while taking alcohol or drugs<br>• Impact of community, partners or others individuals' feelings about the ring

Study Product Discontinuation [*Only for participants discontinuing product early, otherwise skip to question 26*]	
**I know that you stopped using the ring. Before we finish our discussion, I'd like to talk about that experience...**	
**24. Can you tell me what happened?**<br>*Possible probing topics:*<br>• Timing and circumstances of stopping ring use (including sero-conversion)<br>• Aspects of participant's personal life/circumstances contributing to product discontinuation, such as major life events (travel/relocation, work, marriage etc.) or her relationship with her current partner<br>• Any other factors contributing to these circumstances<br>• Feelings about ring discontinuation	
**25. [For HIV sero-convertors only] If it's okay with you, I'd like to talk more about your HIV sero-status…What in your view may have contributed to you getting HIV?**<br>*Possible probing topics:*<br>• Discuss any particular time or event where participant thinks she may have gotten HIV<br>• Discuss any recent changes in personal life, behaviors or circumstances<br>• Potential source of infection<br>• Perceived role of study participation on risk of HIV<br>• Perceived effects (beneficial and toxic) of the ring<br>• Perceptions that participant received active or placebo ring	
**Wrap-up**	
**26. We have talked about many things today. Before we finish, I wonder if you have any questions for me or if you have any additional comments about your experience in ASPIRE or about the discussion today?**	**22. We have talked about many things today. Before we finish, I wonder if you have any questions for me or if you have any additional comments about your experience in ASPIRE or about the discussion today?**

We've now reached the end of our discussion. Thank you for taking the time to talk to me today and share your opinions. We truly appreciate your willingness to participate and discuss your experience with us.

[*Interviewer should answer any unanswered questions, and provide references as needed or clarify any misconceptions at this time*.]
