# Supplementary material for: Experiences with simultaneous use of contraception and the vaginal ring for HIV prevention in sub-Saharan Africa
Source: BMC Womens Health. 2021 Apr 23;21:175. doi: 10.1186/s12905-021-01321-5 (PMC8067655; doi:10.1186/s12905-021-01321-5)
Supplement: Supplementary file 2 — Additional file 2. Focus group discussion topic guide. [file 12905_2021_1321_MOESM2_ESM.pdf]

MTN-020

Focus Group Discussion (FGD) Topic Guide

INSTRUCTIONS for the Facilitator: How to use the FGD Guide

1. There are two levels of questions:

   - Primary discussion questions: appear in **bold** text. They address the topics that you as the facilitator must ask and discuss with participants. The questions are suggestions for getting the discussion going. You are not required to read them verbatim, but they are written to ensure some consistency across FGDs. You may adapt the questions and/or ask them in a different order, depending on how the discussion develops. However you will have to ensure that by the end of the interview, all the topics and key themes have been covered.

   - Probing topics: are indicated with a bullet. If you find that the participants provide little information in response to the primary question, these probing topics may be used to encourage further discussion. You are not required to cover every topic listed. So, depending on what has already been discussed, and the FGD context, you may ask these probes or not.

2. *Instructions/suggestions to facilitator are in italics and [brackets].*

3. The FGD guide is not meant to be used to take notes. Rather, you should use the separate notes form, where you will also insert your initials, the PTIDs of all participants, as well as the date, start and end time of the discussion.

Before starting the FGD, the facilitator reminds the group of:

- The purpose of the FGD
- Ground rules for FGD (per study SSP), including importance of confidentially and use of pseudonyms
- That tape recording will now begin

[*Facilitator starts the tape recorder and reads:*] **As you know from your informed consent, this FGD will be tape recorded today. Before we start, please verbally indicate that you are aware that we are tape recording this session and that it is okay with you** ***[be sure to get a verbal okay from all members of the focus group before continuing].***

**Community**
**First I want to talk about your community where you live…**
**1. How does your community view HIV?**<br>*Possible probing topics:*<br>• Attitudes towards people/women/men/children living with HIV<br>• Attitudes towards HIV prevention, including the use of condoms<br>• Attitudes towards HIV testing in general, including women who get tested (either in trials, in clinics, or mandatory testing when pregnant)<br>• Attitudes towards HIV prevention research
**2. How did people in your community talk about ASPIRE?**<br>*Possible probing topics:*<br>• Knowledge of study and the ring<br>• Positive and negative attitudes/comments/rumors about study or ring<br>• Positive and negative attitudes/comments/rumors about ASPIRE participants<br>• Effects of attitudes/comments/rumors on participants
**Motivation to Join in ASPIRE**
**3. In general how do you think women who choose to join research studies are different from other women in their communities?**<br>*Possible probing topics:*<br>• Describe what kind of women come to get screened in a research study<br>• Differences in their personal relationships, including with male partners and with family members<br>• Differences in their HIV risk (e.g. awareness of partner having other partners or herself having multiple partners)<br>• Differences in their resources<br>• Differences in their personalities
**4. Why would some women choose *not* to participate in ASPIRE?**<br>*Possible probing topics:*<br>• Requirements of participating in trial, such as clinical procedures/testing<br>• Aspects of participants' personal lives (i.e. work commitments, fear or lack of support from partners, etc.)<br>• Aspects of the community/the place where the participants live (i.e. transport, safety, distrust of research, rumors, fear of abuse, violence, rape, HIV stigma, etc.)<br>• Concerns about becoming a research participant, testing an investigational product
**Disclosure and Influence of Others**
**5. Who did women in ASPIRE talk to about their participation and who did they hide it from?**<br>*Possible probing topics:*<br>• Types of people disclosed to and why<br>• Types of people *not* disclosed to and why, including role of fear or experience of violence
**6. What was the reaction of people who women talked to about ASPIRE?**<br>*Possible probing topics:*<br>• Initial reaction and changes in opinions/attitudes – who was supportive, who was discouraging

- Specific actions/reactions of people that made participant feel supported/judged/discouraged
- How reactions of people differed from what the participant anticipated/expected their reactions to be
- Feelings/influence of these people's reactions on participants

Experience in ASPIRE

Now we are going to talk more about women's specific experiences being research participants in ASPIRE…

7. Tell me what it was like to be a research participant for ASPIRE.

Possible probing topics:

- Benefits and challenges of participation
- Expectations of staff and/or the trial
- Experiences resulting from being a participant, including during study visits or at the clinic
- Effects of study on daily life (activities, home/responsibilities, schedule) for participant, partner(s), and others (peers/friends, family members)
- Changes in relationships, including how study participation influenced decision-making and communication around HIV prevention method use, contraceptive use, sexual health and STI treatment needs (if applicable) with male partners
- Changes in social life or alcohol/drug use

8. What were the relationships like among fellow participants in ASPIRE?

Possible probing topics:

- Level of interaction, including discussions in waiting area and outside of the clinic setting
- General feelings about/attitudes towards other participants
- Attitudes of other participants towards the study and ring
- Influence of other participants on study experience/ring use, including discouragement or support

9. Can you describe how participants were treated by study staff in ASPIRE?

Possible probing topics:

- Feelings about different types of staff
- Perceived level of support and what contributed to feeling supported
- Perceived level of judgment and what contributed to feeling judged
- Influence of staff on experience in ASPIRE/use of the ring
- Influence of feedback based on ring appearance, or site level adherence measurements on relationship with staff

10. What were participants' attitudes towards workshops, meetings, and other social activities at the clinic?

Possible probing topics:

- What was useful or not useful (e.g. clinical ring use inspections, adherence counseling or other site specific activities, such as male partner engagement events, morning meetings, adherence scenarios, adherence workshops, educational sessions, etc.)
- Impact of these activities on visit attendance and ring use
- Perceived peer or staff pressure about reporting behaviors while in the trial

Experience using the ring

11. Tell me what it was like for study participants to use the ring.

Possible probing topics:

- Physical experiences of ring use during daily activities
- Side effects or other effects of the ring
- Likes and dislikes of ring and ring use

- Ease or difficulty of use
- Experiences of expulsion or removal, including circumstances and responses

12. How were participants' male partners involved in the study and ring use?

Possible probing topics:

- Level and ways male partners were involved, including in decision-making around participation/ring-use, and why
- Influence of partner attitudes and involvement on experience or ring use
- Change in partner attitude about study participation/ring use over time
- Attitudes towards activities geared towards couples vs. male partners only
- Communication around other health related issues raised by the study

13. How did using the ring affect participants' sexual lives?

Possible probing topics:

- Her or partner's feeling of/ towards the ring during sex, both initially and later in the study
- Positive and negative physical or emotional changes with sex
- Changes in sexual practices, intimacy, sexual partners, frequency, or reasons for having sex, etc.
- Overall occurrence of ring removals before or during sex

14. How did the monthly HIV testing in ASPIRE influence participants' use of the ring?

Possible probing topics:

- Change in level of worry about HIV and why
- How worries influenced decision to stay in ASPIRE and use the ring
- How worries influenced other HIV prevention behaviors

15. How did participants' contraceptive use during ASPIRE influence their use of the ring?

Possible probing topics:

- Changes in contraceptive methods used
- Experience of and concerns around contraceptive side effects (including changes in menstrual cycle and symptoms)
- Impact of experience or concerns around contraceptive side effects on participation/ring use
- Concerns around fertility associated with contraceptives and why
- Knowledge/attitude/feelings of participant and male partners toward use of or changes in contraceptive use

16. What happened to condom use while participants were in ASPIRE?

Possible probing topics:

- Changes in patterns of condom use (study condoms or other condoms), including ability to negotiate their use with partners
- Reasons for changes, if any
- Experience for her and partner managing/negotiating use of both condom and ring simultaneously

17. How did participants' ring use interact with menses and practices to clean or tighten the vagina?

Possible probing topics:

- Preference for use/non-use of ring during menses and why
- Perception of ring's impact on menses and/or fertility
- Perception of hygiene, cleanliness/dirtiness of ring related to use during menses
- Change in vaginal practices and their relation to the study or ring use

Recommendations and Feasibility of Future Use

We are coming towards the end of our discussion. Before we finish, I would appreciate your recommendations and thoughts to help us with future work…[*Only ask these questions if study results*

are not yet publicly known, otherwise skip to question 20]

18. If the ring is proven effective in the future, who do you think would be most likely to use it?

Possible probing topics:

- Types of women most likely to use ring (e.g. girls, older women, married/unmarried, sex workers, "promiscuous" women, women who occasionally drink alcohol or who use alcohol frequently, women who experience violence at home)
- Types of women that community members would expect to use the ring (e.g. see above)

19. If the ring is found to protect from HIV, what factors would influence your decision to use it or not?

Possible probing topics:

- Perception of whether you are at risk of HIV
- How partners are made aware of and involved in encouraging ring use, including their reactions to and experience with the ring
- Ability to participate in the open-label extension study
- Ability to use and interest in using the ring compared to other available alternatives
- Issues around cost, access, storage, disposal, discreetness
- Issues around physical ring appearance, such as color, texture, and size
- Availability of single vs. multi-purpose ring (i.e. for HIV and FP)
- Concerns about *short term vs. long term use on fertility/health or hygiene*
- Concerns about wearing a device in the vagina versus being exposed to the drug
- Concerns about experiencing social harms, violence or rape
- Impact of perceived community or others individuals' attitudes towards the ring
- Reaction/attitudes of people towards women who would use the ring and reasons for these reactions – concerns about promiscuity, stigma, etc.

Stopping Early for Futility, Efficacy, or Due to Harm [*Only ask these questions if either the ASPIRE or Ring (i.e. IPM 027) study is stopped early for any reason, otherwise skip to question 24*]

20. What did you hear about the [ASPIRE/Ring] study being stopped early?

Possible probing topics:

- Source(s) of information
- Understanding of results
- Who was talked to and who have you not talked to about the study stopping early
- Other people's reaction to this, including people they disclosed to, other participants, etc.

21. [*Ask only if one of the studies is stopped for futility*] How do you feel about the fact that in the [ASPIRE/Ring] study, the ring didn't show protection against HIV?

Possible probing topics:

- Change in feelings towards study, ring, or research in general
- Why it didn't show protection

22. [*Ask only if one of the studies is stopped due to efficacy*] How do you feel about the fact that the ring in the [ASPIRE/Ring] study was found to protect against HIV?

Possible probing topics:

- Change in feelings toward study or ring
- Feelings about/understanding of partial efficacy (i.e. that the ring may not protect women 100% of the time), how this would influence ring use, how this would influence condom negotiation with partners
- Interest in participating in open label ring extension study, why or why not
- Thoughts about use of ring outside the research study context

Staff Initials / Date

• Potential change in other HIV prevention behaviors, such as condom use or HIV testing
**23. [*Ask only if one of the studies is stopped due to harm*] How do you feel about the fact that the ring in the [ASPIRE/Ring] study was found to be harmful?**<br>*Possible probing topics:*<br>• Change in feelings toward study or ring<br>• Change in feelings toward research in general
**Wrap-up**
**24. We have talked about many things today. Before we finish, I wonder if you have any recommendations for what could we have done to improve your experience in the ASPIRE study or in using the ring?**<br>*Possible probing topics:*<br>• Study visits, study procedures, clinic<br>• Interaction with staff, counseling sessions<br>• Male partner involvement and/or couples counselling<br>• Providing information or education to others, including facilitated disclosure to others such as partners or family members<br>• Instructional materials, or how the instructions were provided<br>• Changes in physical design of ring, insertion, removal method, frequency of ring replacement

We've now reached the end of our discussion. Do you have any questions for me or any additional comments about your experience in ASPIRE? Thank you for taking the time to talk to me today and share your opinions. We truly appreciate your willingness to participate and discuss your experience with us.

[*At this time, the facilitator should answer any unanswered questions and clarify any misconceptions, or refer participants to someone who can*.]
